# MYC targeting by OMO-103 in solid tumors: a phase 1 trial

**DOI:** 10.1038/s41591-024-02805-1

**Published:** 2024-02-06

**Authors:** Elena Garralda, Marie-Eve Beaulieu, Víctor Moreno, Sílvia Casacuberta-Serra, Sandra Martínez-Martín, Laia Foradada, Guzman Alonso, Daniel Massó-Vallés, Sergio López-Estévez, Toni Jauset, Elena Corral de la Fuente, Bernard Doger, Tatiana Hernández, Raquel Perez-Lopez, Oriol Arqués, Virginia Castillo Cano, Josefa Morales, Jonathan R. Whitfield, Manuela Niewel, Laura Soucek, Emiliano Calvo

**Affiliations:** 1 Vall d’Hebron Institute of Oncology, Barcelona, Spain; 2 Peptomyc S.L., Barcelona, Spain; 3 START Madrid-FJD-Hospital Fundación Jiménez Díaz, Madrid, Spain; 4 START Madrid-CIOCC-Centro Integral Oncológico Clara Campal, Madrid, Spain; 5 Institució Catalana de Recerca i Estudis Avançats, Barcelona, Spain; 6 Department of Biochemistry and Molecular Biology, Universitat Autònoma de Barcelona, Bellaterra, Spain

**Keywords:** Oncogenes, Targeted therapies

## Abstract

Among the ‘most wanted’ targets in cancer therapy is the oncogene MYC, which coordinates key transcriptional programs in tumor development and maintenance. It has, however, long been considered undruggable. OMO-103 is a MYC inhibitor consisting of a 91-amino acid miniprotein. Here we present results from a phase 1 study of OMO-103 in advanced solid tumors, established to examine safety and tolerability as primary outcomes and pharmacokinetics, recommended phase 2 dose and preliminary signs of activity as secondary ones. A classical 3 + 3 design was used for dose escalation of weekly intravenous, single-agent OMO-103 administration in 21-day cycles, encompassing six dose levels (DLs). A total of 22 patients were enrolled, with treatment maintained until disease progression. The most common adverse events were grade 1 infusion-related reactions, occurring in ten patients. One dose-limiting toxicity occurred at DL5. Pharmacokinetics showed nonlinearity, with tissue saturation signs at DL5 and a terminal half-life in serum of 40 h. Of the 19 patients evaluable for response, 12 reached the predefined 9-week time point for assessment of drug antitumor activity, eight of those showing stable disease by computed tomography. One patient defined as stable disease by response evaluation criteria in solid tumors showed a 49% reduction in total tumor volume at best response. Transcriptomic analysis supported target engagement in tumor biopsies. In addition, we identified soluble factors that are potential pharmacodynamic and predictive response markers. Based on all these data, the recommended phase 2 dose was determined as DL5 (6.48 mg kg^−1^).

ClinicalTrials.gov identifier: NCT04808362.

## Abstract (web summary)

In this first-in-human phase 1 dose-escalation trial of OMO-103, a MYC inhibitor, in patients with solid tumors, treatment was safe and showed preliminary clinical activity along with demonstrated target engagement and identification of potential pharmacodynamic markers.

## Main

In human cancers MYC is a frequently deregulated oncogene and a ‘most wanted’ target in cancer therapy^1^. It works as a pleiotropic transcription factor coordinating transcriptional programs involved in cell proliferation, cell growth, metabolism, apoptosis and immune suppression^2^. Under physiological conditions these activities are generally transient and associated with tissue regeneration programs. However, in cancer, MYC and all its corollary functions are relentlessly engaged by upstream oncogenic signals to be continuously permissive for cancer initiation and/or maintenance^3^. It has been suggested that, under these aberrant conditions, MYC could function as a global amplifier increasing the output at all active promoters, leading to hypertranscription in cancer cells^4^. All these factors have pointed towards MYC as an excellent therapeutic target but several technical difficulties, especially the intrinsically disordered nature of the protein, have so far prevented the development of a clinically viable MYC inhibitor^1,5^. Here we report the results of a first-in-human phase 1 clinical trial to assess the safety, pharmacokinetics (PK) and preliminary signs of activity of OMO-103, a first-in-modality anti-MYC miniprotein. OMO-103 is a drug based on Omomyc, a MYC dominant negative initially designed and published in 1998 as a laboratory tool to study MYC perturbation^6,7^, then later used to model MYC inhibition and its marked therapeutic potential in different mouse models of cancer, where it also showed safety and tolerability^6,8–10^. To summarize, Omomyc interferes with MYC dimerization to its obligate partner MAX and inhibits their interaction with the consensus DNA-binding site, the E-box sequence. Indeed, Omomyc sequesters MYC in protein dimers unable to bind DNA while also forming both homodimers and heterodimers with MAX, which occupy the E-boxes with transcriptionally inactive protein complexes, preventing the transcription of bona fide MYC targets^6,7,11^. Omomyc was previously expressed genetically to show that it could prevent tumorigenesis and even eradicate tumors in multiple cancer models, regardless of their driving oncogene or tissue of origin^6,8–10,12–14^.

The more recent discovery of unexpected cell-penetrating properties of the purified Omomyc miniprotein led to its development as a pharmacological tool that demonstrated therapeutic efficacy both in vitro and in mouse models of non-small cell lung cancer (NSCLC)^15^ and triple-negative breast cancer^16^ and opened the way for its clinical development^17^. In these contexts, Omomyc showed safety and efficacy both upon intranasal and intravenous administration and induced shutdown of MYC transcriptional programs and reprogramming of the tumor microenvironment^18^, recapitulating several key features of expression of the Omomyc transgene^15,16^. Its therapeutic impact was reported in both primary tumors and metastases^16^, alone and in combination with standard-of-care chemotherapy (that is, paclitaxel)^15,16^.

In these preclinical models, the degree of response to Omomyc correlated only with its level of expression and not with MYC levels^6,14,16^.

Here we report the results of a dose-escalation phase 1 study in allcomers solid tumors, showing safety and preliminary signs of drug activity of OMO-103 under different oncological indications, supported by target engagement and immune-related biomarkers.

## Results

### Dose escalation and safety study

**Fig. 1:**
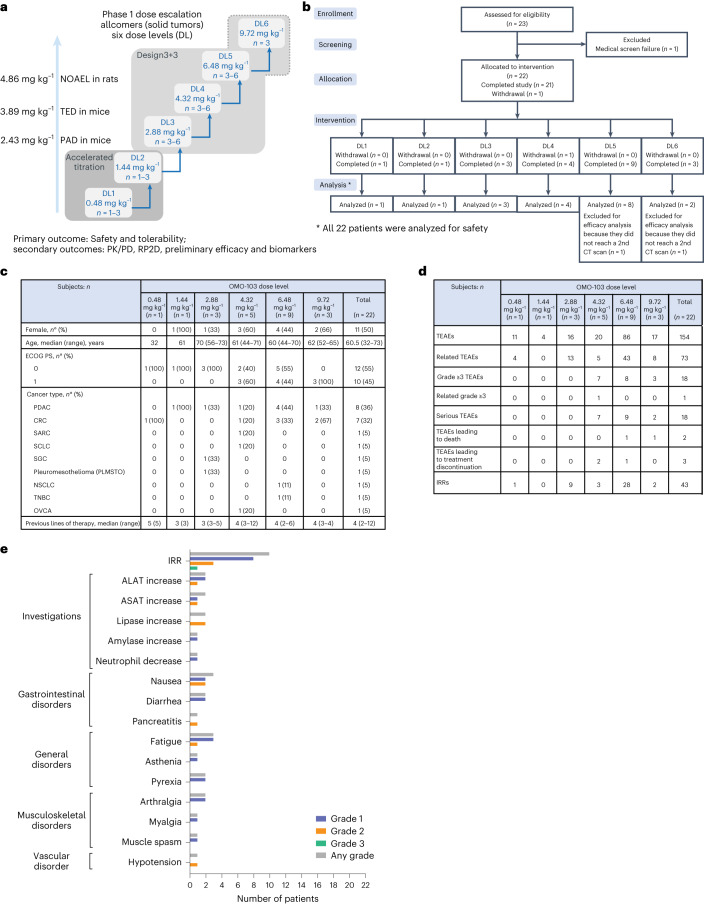
Overall trial design and safety. **a**, Schematic of dose-escalation design. PAD, pharmacologically active dose; TED, therapeutically effective dose; NOAEL, no observed adverse effect level; *n*, number of patients. **b**, CONSORT flow diagram. **c**, Demographic and baseline characteristics. ECOG PS, ECOG performance status. **d**, Overall safety. TEAE, treatment-emergent adverse event. **e**, TRAEs (*n* = 22). System organ class and symptoms are shown. SCLC, small cell lung cancer; NSCLC, non-small cell lung cancer; TNBC, triple-negative breast cancer; ALAT, alanine aminotransferase; ASAT, aspartate aminotransferase. Source data

**Extended Data Fig. 1:**
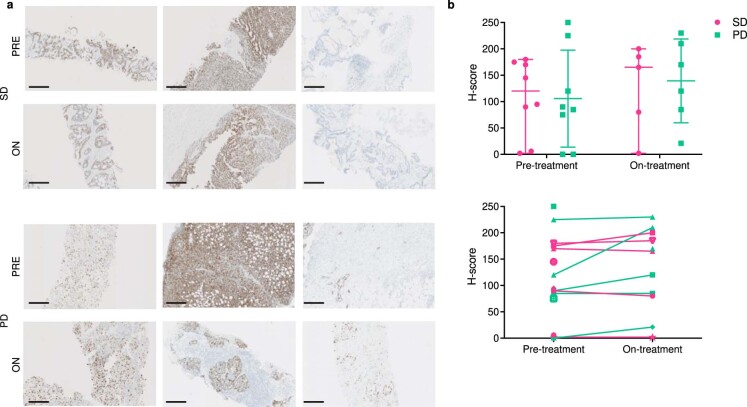
MYC levels in patient biopsies. **(a)** Representative images of pre- and on-treatment biopsies from stable and progressive disease patients. **(b)** Quantification of MYC protein levels determined by immunohistochemistry and given as H-score, a semi-quantitative assessment that takes into account the intensity (graded as 0, 1+, 2+, or 3+ for each cell) and percentage of positive cells according to the formula: H-score = [1 × (% cells 1+) + 2 × (% cells 2+) + 3 × (% cells 3+)]. n = 16 patients at pre-treatment. n = 11 patients at on-treatment. In the upper plot, the median value and 95% confidence intervals are shown. In the lower plot, the variation in H-score between pre- and on-treatment is shown per patient, when possible. A mixed-effects analysis with Bonferroni’s correction for multiple tests was used.

The study was carried out from May 2021 until October 2022. The first patient was enrolled on 4 May 2021 and the last on 4 April 2022; 22 patients were enrolled and treated at six DLs (1–6) (Fig. 1a,b). The patients included had a wide range of metastatic solid tumors and had received a median of four previous lines of treatment, ranging from two to 12 (see Fig. 1c for demographics). Given the lack of correlation between MYC expression and Omomyc effect in preclinical models, neither MYC amplification nor overexpression was used as an inclusion criterion but was analyzed retrospectively. Nine out of the 16 patients (56.25%) that could be evaluated showed >50% MYC^+^ cells at baseline by immunohistochemistry (Extended Data Fig. 1). Only two out of 16 patients (12.5%) showed MYC amplification at baseline but they did not show high MYC levels by immunohistochemistry. Because MYC overexpression has been described as a common mechanism of resistance to multiple drugs^19^, it is likely that the numerous lines of treatment received by patients entering a phase 1 study contributed to its stabilization and/or amplification.

Women and men were equally represented, and an Eastern Cooperative Oncology Group (ECOG) performance status of 0 and 1 was distributed in a similar manner across patients. Median age was 60.5 years. All patients were treated on a weekly basis by 30–45-min intravenous infusion of either 0.48, 1.44, 2.88, 4.32, 6.48 or 9.72 mg kg^−1^ OMO-103 (*n* = 1, 1, 3, 5, 9 and 3, respectively). One cycle of treatment was defined as 3 weeks (that is, three infusions).

An overview of general safety is shown in Fig. 1d, and treatment-related adverse events (TRAEs) in Fig. 1e. The most common TRAEs were mainly grade 1 infusion-related reactions (IRRs), including chills, fever, nausea, rash and hypotension (Fig. 1e and Extended Data Table 1). In total, ten of the 22 patients developed IRRs and six experienced them only once while the other four experienced them multiple times. In seven patients the IRR occurred after the first infusion, starting several hours (2–5) after the end of the infusion and lasting only 1 h or, in the worst case, disappearing overnight. Fifty-eight TRAEs (80.5%) were grade 1, 12 (16.6%) were grade 2 and one (1.4%) was grade 3. The only grade 3 IRR observed was in a patient with ovarian carcinoma (OVCA) who had already received 12 lines of treatment and shown a similar reaction to cisplatin, in addition to intolerance to certain antibiotics (including amoxicillin), and therefore this reaction was considered as neither a dose-limiting toxicity (DLT) nor dose dependent, but patient dependent. Higher DLs (5 and 6) were associated with more IRRs, but those were easily prevented with standard (pre)medication. Four patients had a temporary interruption (about 40 min) of the study drug infusion due to IRR (grade 1). In one patient this happened twice while for the others it occurred only once. Another patient developed a grade 2 IRR and so the study drug was interrupted but, as progression of disease was discovered on the same day, treatment was discontinued permanently. One DLT, a grade 2 pancreatitis, was observed at DL5 in a patient with pancreatic ductal adenocarcinoma (PDAC). This case was discussed with the safety monitoring committee (SMC) and, even though the event did not fulfill the criterion of grade 3, it was decided to classify it as DLT to be on the side of safety. This patient was removed from the study, and recovered quickly and completely. This was the only patient—along with the one with IRR grade 3—to be removed from the study due to a TRAE. In total there were 18 serious adverse events but only one of these was considered drug related (the previously mentioned grade 3 IRR). One patient left the study after only one infusion because he developed brain metastases, which were an exclusion criterion for the study. Two patients showed progression of the disease with eventual fatal outcome (grade 5); both had advanced PDAC.

No maximum tolerated dose (MTD) was reached and, overall, the drug was considered safe and tolerable across all DLs. The recommended phase 2 dose (RP2D) was established at DL5, equivalent to 6.48 mg kg^−1^, based on safety, preliminary antitumor activity, PK, positive target engagement and biomarkers (see below).

### PK studies

**Fig. 2:**
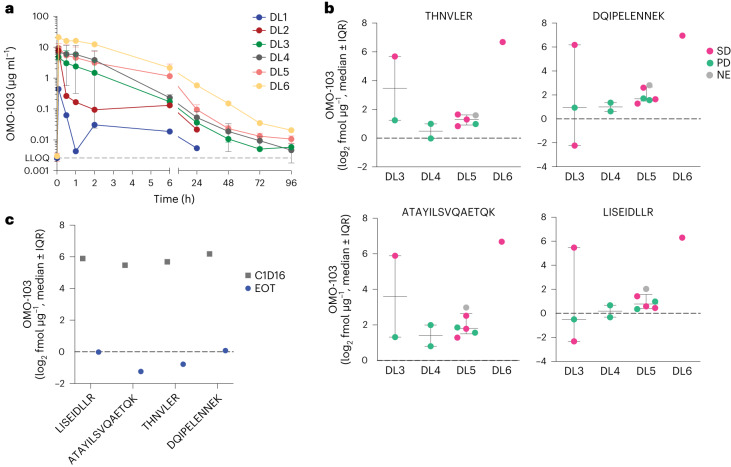
PK of OMO-103 in serum and detection in patient biopsies. **a**, PK profiles at different DLs following a single intravenous infusion of OMO-103 (*n* = 22). LLOQ, lower limit of quantification. Statistical average value and standard deviation are shown. **b**, Quantification of functional Omomyc by MS in FFPE on-treatment biopsies from MYCure patients for DL3–6 following administration of at least three intravenous infusions of OMO-103 (*n* = 12). The sequence of peptides used for detection of the Omomyc protein is indicated on top of the *x* axis. Median value and interquartile range (IQR) are shown. **c**, Results from end-of-treatment (EOT) biopsy of a patient with PDAC from DL3 who remained for >6 months in the study and had received their last infusion 19 days before biopsy, compared with those obtained at cycle 1, day 16 (C1D16). NE, not evaluable. Source data

The PK profile of OMO-103 was studied in serum during dose escalation, and absolute levels of OMO-103 were quantified from tissue biopsies. A summary of the biopsy schedule and samples obtained is shown in Extended Data Table 2. The average PK profile for each DL is shown in Fig. 2a, and descriptive statistics for the parameters are provided in Extended Data Table 3. Nonlinearity was observed above DL5, where the area under the curve (AUC) increased with dose in a nonproportional manner while clearance and volume of distribution diminished after multiple dosing, indicating tissue saturation. Although terminal drug half-life in serum samples was estimated at approximately 40 h, this value is probably an underestimation because PK serum samples were collected only up to 96 h following treatment and OMO-103 showed greater persistence and integrity in tumor tissue compared with that in serum samples. Indeed, using a previously described mass spectrometry (MS) approach based on the detection of four different peptides spanning the entire protein sequence^18^, we were able to detect OMO-103 in patient biopsies even 19 days following the last infusion (Fig. 2b,c).

Antidrug antibodies (ADAs) were also analyzed to evaluate the potential immunogenicity of OMO-103. No ADAs were detected at any DL, even following long-term treatment (up to cycle 12 (C12)), except in one patient at DL5 who also showed elevated levels of rheumatoid factor, often considered a confounding element in ADA assessment^20^ (Extended Data Table 4).

### Antitumor activity

**Fig. 3:**
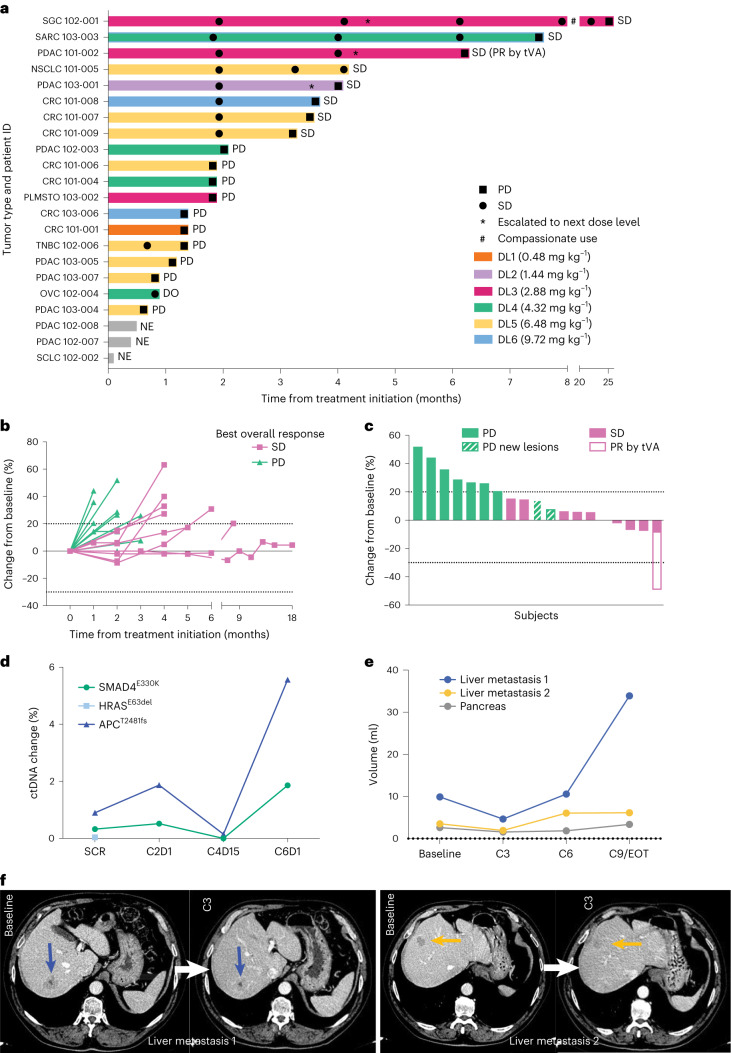
Evaluation of tumor response in all cohorts and time-dependent disease evaluation in one patient with PDAC. **a**, Swimmer plot of 22 enrolled patients. SD, stable disease; PD, progressive disease; DO, dropout; PR, partial response; tVA, Total volumetric analysis. **b**, Spider plot showing changes in target lesion volume from 19 evaluable patients. **c**, Waterfall graph indicating change in volume of target lesions (RECIST v.1.1) at best response of 19 evaluable patients. **d**, ctDNA dynamics of the three somatic alterations identified by Guardant360. **e**,**f**, Baseline, C3, C6 and C9/EOT volume measurement of individual lesions by CT scan (**e**) and representative images (**f**). Blue and yellow arrows indicate lesions quantified in **e**.

Nineteen patients were evaluable for efficacy. Clinical response to the drug was evaluated by computed tomography (CT) scan following 9 weeks of treatment (three cycles) and every 9 weeks thereafter. In addition, the Guardant360 assay, which detects cell-free circulating tumor DNA in blood specimens and evaluates 73 genes, was used as a complementary surrogate measure of tumor response^21^. Twelve patients reached this time point (patient progress throughout the various phases is summarized in Fig. 1b). Eight patients showed stable disease (SD) at best response, at DL ranging from 2 to 6, with duration of disease stabilization thereafter ranging from 35 to 765 days. These patients included two with PDAC, three with colorectal cancer (CRC), one with NSCLC, one with fusocellular sarcoma (SARC) and one with salivary gland carcinoma (SGC). The other ten patients did not reach the prescheduled 9-week CT scan due to either rapid, early progression of disease or had presented with grade 2 pancreatitis or grade 3 IRR as reported above, and hence were withdrawn from the study. A complete evaluation of all patients is shown in Fig. 3 and Extended Data Table 5. The change in percentage of target lesions compared with baseline throughout the study and at best response is shown in Fig. 3b and 3c, respectively. Among these, some standout cases are described in greater detail below.

One patient with PDAC in DL3 received OMO-103 as a fourth line of treatment and remained in the study for approximately 7 months. This patient achieved 8% tumor shrinkage of target lesions according to response evaluation criteria in solid tumors (RECIST) v.1.1 at the first scheduled CT scan, and 83% reduction in ctDNA following 11 weeks on treatment as measured by the Guardant360 assay (Fig. 3d and Supplementary Table 1). Indeed, the variant allele frequency dynamics of the three somatic alterations identified by Guardant360 showed a slight initial increase in SMAD4 E330K and APC T2481fs (from 0.33 and 0.90% to 0.52 and 1.87%, respectively) after 3 weeks, while APC fell markedly under the limit of detection 8 weeks later and SMAD4 decreased to 0.15% variant allele frequency (Fig. 3d). The HRAS variant remained under the detection limit from week 3 onwards and was never again detected; interestingly, when evaluating the change in total radiographic burden of disease (that is, sum of the volume of all lesions) the patient showed a 49% reduction in volume as best response following OMO-103 treatment (Fig. 3e,f).

One patient with fusocellular sarcoma entered the study at DL4 following five previous lines of treatment. He had not benefited from the previous two lines but remained on treatment with OMO-103 for approximately 8 months.

Finally, the patient affected by a SGC (initially treated at DL3 then escalated to DL4) was maintained on treatment under compassionate use following study completion and was stable for approximately 26 months without any AEs.

It is also worth mentioning that the patient with OVCA who abandoned the study because of a grade 3 IRR underwent a CT scan following two cycles with OMO-103, showing stabilization of the disease and a 30% drop in ctDNA (Supplementary Table 1).

Importantly, no correlation was observed between MYC levels (measured by *H*-score) and clinical response (SD versus progressive disease (PD)) (Extended Data Fig. 1).

### OMO-103 treatment is associated with target engagement

**Fig. 4:**
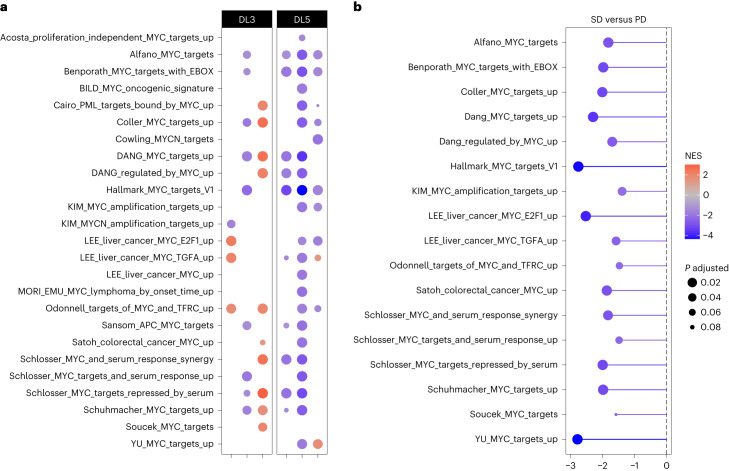
Target engagement analysis by DSP. **a**,**b**, In DL3 and 5, three paired biopsies each were analyzed (*n* = 6 in total). **a**, GSEA comparing the status of each MYC gene set in on-treatment (C1D15)** versus pretreatment biopsies. Normalized enrichment score (NES) is represented by a color scale from red (enriched post treatment) to blue (enriched in pretreatment compared with post), while adjusted *P* value is represented by circle size. For each patient, three to five regions of interest were compiled. **b**, Lollipop graph representing comparison by GSEA of the status of MYC gene sets in samples from patients with SD versus PD. NES is represented by the length of the lollipop and color scale, while the adjusted *P* value is represented by circle size.

Because MYC is a pleiotropic transcription factor, its main function is transcriptional regulation. Previously published work demonstrated that MYC inhibition by Omomyc does not necessarily change MYC levels but consistently impairs its transcriptional activity^6,15,16,22^. Hence, to evaluate target engagement by OMO-103 we performed transcriptomic analysis. To do so, patient biopsies were subjected to digital spatial profiling (DSP), which allowed RNA sequencing of regions of interest and assessment of MYC transcriptional signature by gene set enrichment analysis (GSEA). The study was performed on paired pre- and on-treatment biopsies (Extended Data Table 2). This stringent criterion allowed the analysis of three patients each in DL3 and DL5. DSP results were then cross-compared with published MYC transcriptional fingerprints (Fig. 4a). This analysis revealed a shutdown of multiple MYC transcriptional signatures in one patient in DL3 and in three in DL5, thus correlating the more frequent transcriptional changes with the higher DL. Moreover, patients with SD showed a more profound shutdown compared with those with PD (Fig. 4b).

**Extended Data Fig. 2:**
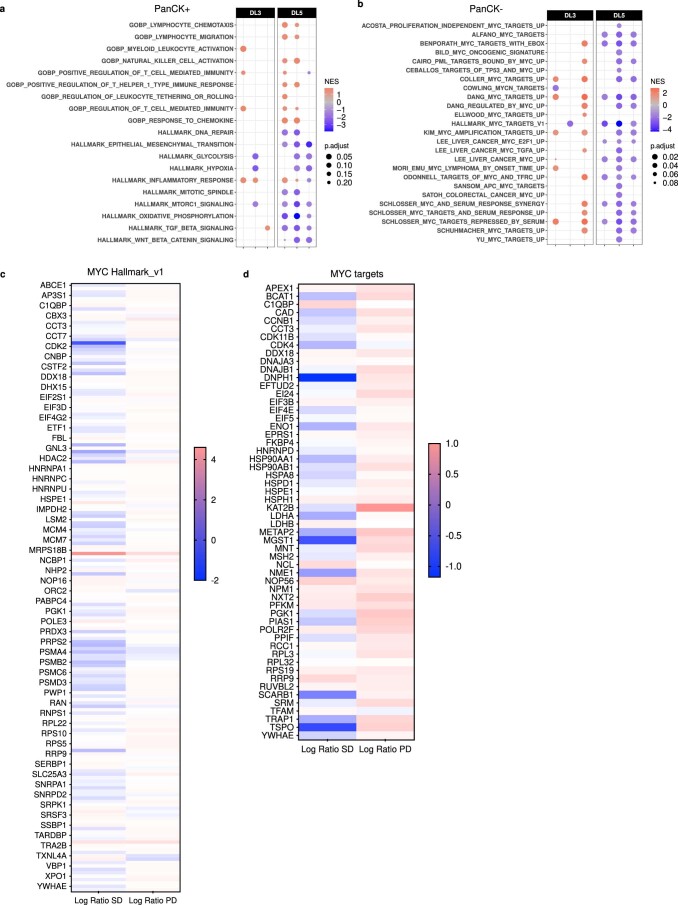
DSP assessment of modulation of additional pathways in the tumor related to cancer and the immune system and target engagement in PanCK- cells, and proteomic analysis of MYC targets. 3 paired biopsies were analyzed in DL3 and 3 in DL5 (n=6 in total). GSEA comparing in on-treatment versus pre-treatment biopsies the **(a)** status of immune- and cancer-related gene sets in the PanCK+ segment and **(b)** status of each MYC gene set in the PanCK- segment. The NES is represented by a color scale from red (enriched post-treatment) to blue (enriched in the pre-treatment compared to the post), while the adjusted p-value is represented by the size of each circle. For each patient, 3–5 regions of interest were compiled. **(c** and **d)** 2 paired biopsies were analyzed in DL3 and 2 in DL5 (n=4 in total) by UltraDeep Protein Profiling. The Log Ratio of SD and PD patients was calculated per protein by dividing on-treatment by pre-treatment values from a subset of **(c)** 179 proteins from the MYC Hallmarks v1 and **(d)** 56 direct targets of MYC. Such values are represented by a color scale from red (upregulated) to blue (downregulated).

In addition, we evaluated the transcriptional impact of OMO-103 on immune- and cancer-related programs (Extended Data Fig. 2a). In line with the immunosuppressive role of MYC in the tumor immune microenvironment, several immune-related gene sets were upregulated following its inhibition by OMO-103, especially those related to T cell-mediated immunity. In regard to MYC signature shutdown, immune-activating and tumor-suppressive phenotypes were more profound in patients from DL5 than from DL3 (Extended Data Fig. 2a).

Of note, the analysis of MYC transcriptional signatures was also performed on pan-cytokeratin (PANCK)-negative (nontumor) cells and showed a MYC fingerprint shutdown also in the tumor microenvironment (Extended Data Fig. 2b), demonstrating that the MYC-inhibitory effect of OMO-103 is both tumor cell autonomous and nonautonomous, reflecting the biological role of MYC in both compartments^2^.

To complement the transcriptomic analysis, ultradeep protein profiling by MS was applied for analysis of MYC target downregulation at the protein level. Despite the limited sample size (a total of eight paired biopsies from two patients each with SD and PD) that prevented statistical considerations, this analysis showed that 110 out of 179 proteins from the MYC Hallmarks gene set were downregulated in patients with SD compared with 35 out of 179 in those with PD (Extended Data Fig. 2c). Similarly, 37 out of 56 direct MYC targets had reduced protein levels following treatment in patients with SD compared with only three out of 56 in those with PD (Extended Data Fig. 2d), once again correlating clinical benefit with more efficient shutdown of MYC targets.

### Predictive and pharmacodynamic liquid biomarkers

As mentioned above, besides having a role in tumor growth, MYC is a well-known modulator of antitumor immune suppression, able to reinstruct the tumor microenvironment towards a tumor-promoting and immune-tolerant phenotype^2,23^. We previously showed that, by inhibition of MYC, Omomyc can reprogram the tumor microenvironment through vascular remodeling and cytokine–chemokine modulation^9,15,23^. Hence, to look for noninvasive biomarkers of response to OMO-103, blood sampling from patients was performed at baseline and during treatment, with serum analyzed by Luminex technology for circulating soluble factors associated with inflammatory processes and potential cytokine release syndrome. Of note, none of the patients showed any sign of such a potential syndrome following treatment.

**Fig. 5:**
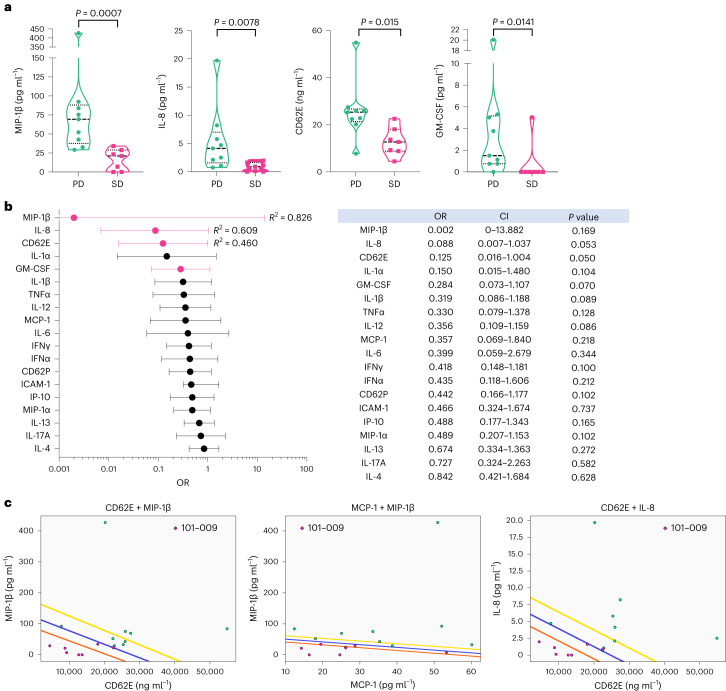
Patients that clinically benefited from OMO-103 showed low baseline levels of MIP-1β, IL-8, CD62E and GM-CSF, with model pairings of different soluble factors providing outstanding outcome predictors. **a**–**c**, Levels of soluble factors were measured in patient serum samples at pretreatment using the Luminex technique. **a**,**b**, Nine patients with PD and seven with SD were included in the analyses. **a**, Patients showing disease stabilization at cycle 3 displayed significantly lower levels of MIP-1β, IL-8, CD62E and GM-CSF compared with those with PD (median shown). A two-sided Mann–Whitney *U*-test, with no adjustment for multiple comparisons, was used for statistical analysis. **b**, Binomial logistic regression analysis of the association of cytokine and chemokine levels at pretreatment with the probability of disease stabilization. ORs of soluble factors in patients are shown in the forest plot (left) and summary table (right); mean and CI are shown. **c**, Soluble factor combination models generated using QLattice technology; data from nine patients with PD and six with SD were used to generate the models. Plots of combination models CD62E + MIP-1β, MCP-1 + MIP-1β and CD62E + IL-8 are shown in relationship to patient response. SD and PD are indicated by pink and green dots, respectively. Colored lines correspond to confidence bands: blue indicates the mean, orange 95% CI and yellow 5% CI. Patient no. 101-009 is marked with a rhombus because their data were not available at the time of generation of the models and were later included. Source data

We initially searched for potential pretreatment biomarkers capable of predicting response to therapy. At baseline, patients that presented with SD at C3 showed significantly lower levels of soluble macrophage inflammatory protein 1-beta (MIP-1β), interleukin-8 (IL-8), CD62 antigen-like family member E (CD62E, also known as E-selectin) and granulocyte-macrophage colony-stimulating factor (GM-CSF) compared with those shown by patients with PD (Fig. 5a). In addition, binomial logistic regression analysis showed that CD62E, IL-8, MIP-1β and GM-CSF had odds ratios (ORs) clearly <1 (CD62E, 0.125 (CI 0.016–1.004, *P* = 0.053, *R*^2^ = 0.46); IL-8, 0.088 (CI 0.007–1.037, *P* = 0.050, *R*^2^ = 0.609); and MIP-1β, 0.002 (CI 0–13.822, *P* = 0.17, *R*^2^ = 0.826)), showing a negative association with SD at C3 (Fig. 5b). It is important to note that, while trends toward significance were observed for CD62E and IL-8, limited sample size and interpatient variability may have contributed to the lack of statistical significance. Of note, in this context MIP-1β showed the strongest association with SD but did not display a statistically significant OR due to the high variability of chemokine levels in the PD group and reduced number of evaluable patients. The trends observed in these data suggest potential associations, warranting further investigation in larger cohorts to validate these preliminary findings.

**Extended Data Fig. 3:**
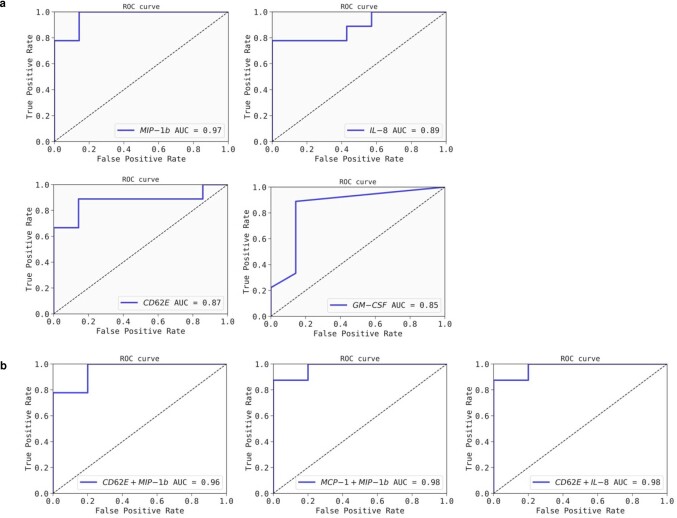
Receiver Operating Characteristic (ROC) curve analysis of the identified soluble factors and predictive combination models. **(a)** Receiver Operating Characteristic (ROC) curve analysis for individual soluble factors to predict response to OMO-103. Individual ROC curve graphs and their corresponding Area Under the Curve (AUC) scores for each individual soluble factor are shown. Each ROC curve illustrates the relationship between the true positive rate and the false positive rate across various classification thresholds. **(b)** ROC curve analysis and ROC-AUC scores of the predictive combination models are shown below. ROC-AUC scores of the combination models are higher than those of the individual factors, suggesting an increased predictive power.

To investigate the predictive power of these identified soluble factors for clinical response to OMO-103, we fitted the values of each factor to a univariate logistic regression model. The performance of these models was evaluated with receiver operating characteristic (ROC) curve analysis. ROC–AUC values were 0.97 for MIP-1β, 0.89 for IL-8, 0.87 for CD62E and 0.85 for GM-CSF, all >0.8, indicating that these models represent excellent SD outcome predictors (Extended Data Fig. 3a).

Most interestingly, subjecting the Luminex data to QLattice technology enabled us to identify a distinct signature predictive of disease stabilization in response to OMO-103 and independent of oncological indication or previous treatment. Such a signature encompasses four of the soluble factors identified as significantly lower at baseline for patients with SD. These factors generate three independent models of prediction (Fig. 5c) that include the combination of two soluble markers. Again, ROC curve analysis suggested that these three combination models are outstanding predictors of SD outcome and that their predictive power is improved compared with that of their individual models (CD62E + MIP-1β, AUC = 0.96; MCP-1 + MIP-1β, AUC = 0.98; CD62E + IL-8, AUC = 0.98; Extended Data Fig. 3b).

**Fig. 6:**
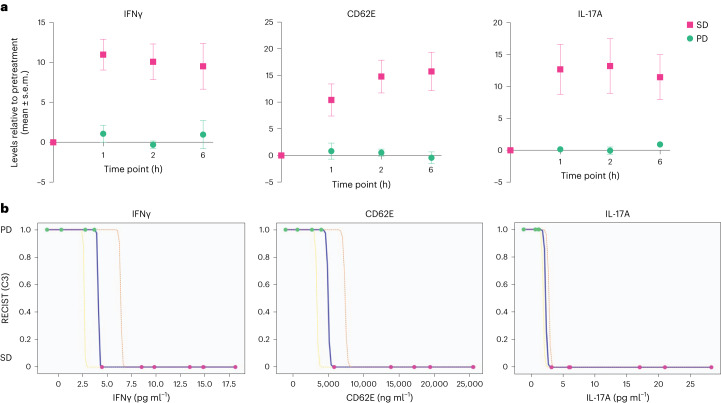
Following OMO-103 infusion, patients with disease stabilization at C3 showed significantly increased levels of IFNγ, CD62E and IL-17A, which can be used to identify patients with SD or PD. **a**,**b**, Levels of different soluble factors were measured in patient serum samples taken at different time points following OMO-103 treatment and were determined using the Luminex technique. Four and six patients with PD and SD, respectively, were included in the analysis. **a**, At the onset of C3, patients with disease stabilization at the following CT scan showed a significant increase in levels of IFNγ (*P* = 0.000148), CD62E (*P* = 0.000338) and IL-17A (*P* = 0.0013) following OMO-103 infusion compared with patients with PD. Mean and s.e.m. are shown. A two-sided Welch’s *t*-test was used with Bonferroni correction to adjust for multiple comparisons. **b**, Individual IFNγ, CD62E and IL-17A models generated with QLattice technology. Maximum serum levels of the indicated cytokines were used to generate the models. Serum levels of patients with PD and SD are indicated as 1 and 0, respectively. Colored lines correspond to confidence bands: blue indicates the mean, yellow 5% CI and orange 95% CI. Source data

Subsequently, following the same process and rationale, we sought to determine a pharmacodynamic signature of response during treatment. In this case we found three soluble factors transiently induced following infusion with OMO-103 starting from C3 onwards (Fig. 6a). This signature had already been detected ~2–3 weeks before the predefined 9-week CT scan assessment and was observed only in patients who then showed SD while it was absent in those with PD. Indeed, all patients showing SD following 9 weeks of treatment displayed significantly increased levels of interferon-γ (IFNγ), CD62E and interleukin-17A (IL-17A) at several time points following OMO-103 infusion. In contrast, in patients with PD the levels of these markers remained completely stable (Fig. 6a). Of note, the transient increase in levels of these soluble factors was observed at the onset of C3 in all patients showing SD, except in patient no. 102-001, who nevertheless proceeded to evidence increase at C6. This signature was independent of oncological indication or previous treatments (Supplementary Table 2).

**Extended Data Fig. 4:**
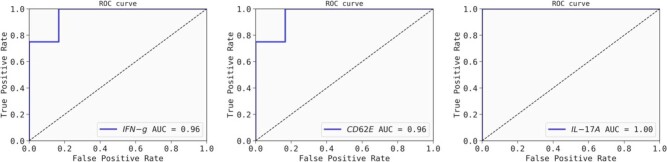
Receiver Operating Characteristic (ROC) curve analysis of the individual pharmacodynamic models. Receiver Operating Characteristic (ROC) curve analysis of the individual pharmacodynamic models to identify SD vs PD patients upon OMO-103 treatment. Individual ROC curve graphs and ROC-AUC scores for each individual model are shown. Each ROC curve illustrates the relationship between the true positive rate and the false positive rate across various classification thresholds.

As described above, further analysis of these data with the QLattice technology generated one independent model for each of the soluble factors capable of efficiently distinguishing patients with SD and PD (Fig. 6b). Once again, ROC curve analysis showed that the AUC for these three models was very close to 1 (IFNγ, AUC = 0.96; CD62E, AUC = 0.96; and IL-17A, AUC = 1; Extended Data Fig. 4).

**Extended Data Fig. 5:**
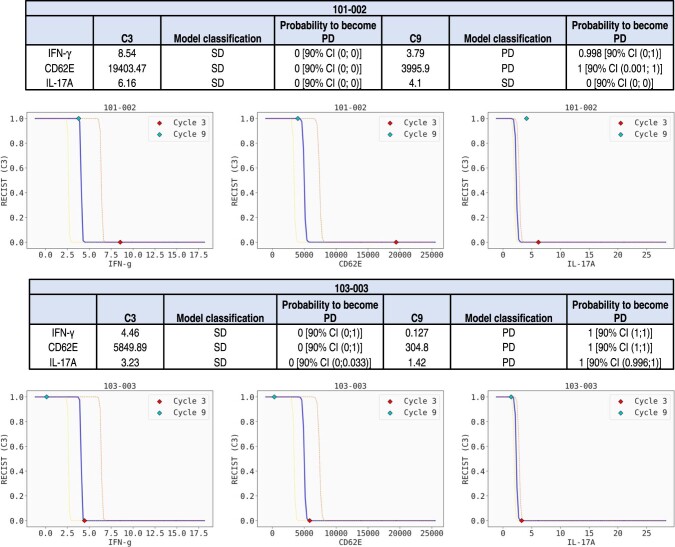
The pharmacodynamic signature can be associated to maintained SD and is lost upon disease progression. The levels of IFN-γ, CD62E and IL-17A are not transiently increased after OMO-103 infusion for patients 101-002 and 103-003 at C9, time point at which they displayed PD as per RECIST criteria. Tables indicate the values relative to pre-dose at the maximum peak, how the model classifies them in terms of SD or PD, and the probability to become PD according to their maximum relative levels. Graphs of the three independent models show the relative values of each soluble factor at C3 and C9. C3 is marked as a red dot and C9 as a yellow dot. CI: confidence interval.

Notably, the transient increase in serum levels of IFNγ, CD62E and IL-17A following treatment is associated with maintenance of SD; once patients start to progress, their peak levels decrease or even disappear (Extended Data Fig. 5). This was seen in patient nos. 101-002 and 103-003 at C9, the time point at which they transited from SD to PD according to RECIST. Note that none of the patients showed ADAs at any point during treatment, suggesting that this cytokine signature is a specific antitumor immune response (Extended Data Table 4).

## Discussion

The most deregulated oncogene in human cancers is MYC but it has remained an elusive target in cancer biology for decades. Here we present what, to our knowledge, is the first successful phase 1 clinical trial validating the safety and tolerability of a first-in-modality direct MYC inhibitor, OMO-103, derived from the most extensively characterized MYC inhibitor to date^6,15,17^. OMO-103 showed safety, causing mainly grade 1 side effects in patients—mostly infusion-related reactions of low grade that are often demonstrated by biologics. The drug also showed suitable PK properties up to the recommended dose, with low or absent immunogenicity.

In addition, the study indicated encouraging signs of drug activity based on both clinical response and assessment of molecular target engagement and biomarkers of response. With regard to clinical activity it is important to highlight that, even though there was no objective response to treatment according to RECIST criteria, at least one patient demonstrated 49% tumor reduction by total tumor burden volume quantification and half of the patients (all showing very resistant and advanced disease) benefited from OMO-103, undergoing disease stabilization. Notably, one patient received treatment under compassionate use after the study was closed and benefited from it for approximately 26 months.

Importantly, drug activity was also supported by target engagement as demonstrated by the shutdown of bona fide MYC-driven transcriptional signatures in patient biopsies. Although this analysis was performed on a limited number of samples it should be noted that, to our knowledge, this is the first time that such an effect has ever been shown in patients with any experimental anti-MYC therapeutic^5,17^.

Taking advantage of the double role of MYC in both intra- and extracellular programs of tumorigenesis, we were also able to identify soluble biomarkers of response to MYC inhibition in serum samples. Our findings suggest both a predictive and pharmacodynamic signature. These can be detected by liquid biopsies, and include well-known soluble factors involved in activation of the immune response. Following treatment with OMO-103, patients showing disease stabilization presented transiently increased levels of IFNγ, CD62E and IL-17A. IFNγ is a pleiotropic cytokine with well-known antitumor activity, mainly promoting antigen presentation and activating cytotoxic immune cells. In addition, it also has direct cancer cell-specific antitumor effects such as inhibition of proliferation and induction of apoptosis^24,25^. Based on these properties, IFNγ treatment is considered potentially useful as an adjuvant immunotherapy^26^. In contrast, the exact role of IL-17 in tumor immunity is still under debate, mainly due to the wide variety and high plasticity of IL-17-producing cells. However, the secretion of IL-17 along with other effector cytokines such as IFNγ can exert potent antitumor effects boosting tumor-specific T cell responses through enhanced dendritic and cytotoxic T cell recruitment to the tumor bed^27^. Finally, CD62E is expressed on cytokine-activated endothelial cells and mediates leukocyte migration into inflamed tissues, a fundamental prerequisite for the entry of antitumor effector cells to the tumor site^28^. These results are in line with those obtained from differential gene expression analysis of tumor biopsies, where several gene sets related to T cell immunity and migration were upregulated following OMO-103 treatment. Notably, all three soluble factors have been associated with wound healing and wound resolution, an interplay often defective in cancer and metastasis^29–32^, strictly related to MYC biology^33^. The transient increase of these proinflammatory soluble factors that occurs only in patients with SD is distinct from a chronic inflammatory response and may indicate an antitumor immune response triggered by OMO-103 treatment. Importantly, none of the patients showing the pharmacodynamic signature presented ADAs.

In conclusion, we believe that these findings and the safety profile of OMO-103 encourage further investigation of its clinical activity and safety in specific indications. In addition, a combination of MYC inhibition with other treatments—chemotherapy, personalized medicine and immunotherapy—could increase their efficacy both in contexts where MYC is not overexpressed as well as where it is amplified or overexpressed (a common mechanism of drug resistance^19^).

Among the limitations of this clinical study, it did not include MYC amplification or overexpression as inclusion criteria, based on the concept that MYC addiction is not necessarily dependent on its absolute levels but more on its tonic, deregulated signaling^34^. In addition, we saw no correlation between Omomyc efficacy and MYC levels in preclinical studies^6,14,16^. Nevertheless, the question remains as to whether varying MYC expression levels might reveal a different sensitivity to OMO-103, especially in earlier lines of treatment. Future clinical studies will hopefully shed light on this matter.

The identification of pharmacodynamic and predictive biomarkers is based on a limited and heterogenous number of patients, with very different previous lines of treatment (as is inherent in any phase I study in oncology), and hence their validity and potential antitumorigenic role require further testing in a larger and more homogenous patient population. The identified signatures are indeed currently being tested in a new clinical study with OMO-103 in combination with standard-of-care (SoC) chemotherapy (ClinicalTrials.gov identifier: NCT06059001), where the predictive signature is being used for patient selection.

## Methods

### Study design and patient population

MYCure (NCT04808362) is a first-in-human, open-label, multicenter, phase 1 dose-escalation study of six DLs evaluating the safety of OMO-103 across solid tumors conducted at three sites in Spain.

Complete and signed written informed consent was obtained from patients for inclusion in the study. There was no remuneration for participation in the trial but there was compensation for travel expenses.

The study included histologically or cytologically proven advanced solid tumors for which there was no curative therapy, that had progressed on SoC treatment, were intolerant to it or had no available SoC or for which SoC was unacceptable. Patients had to demonstrate measurable disease according to RECIST v.1.1 criteria^35^ as demonstrated by CT/magnetic resonance imaging and documented progression on or following the last line of therapy. They also had to present an ECOG performance status of up to 1, life expectancy of ≥12 weeks and adequate organ function.

For patient demographics please refer to Fig. 1c. Women and men were equally represented in the study. Sex of participants was determined based on self-reporting.

OMO-103 was administered by weekly intravenous infusion over 30–45 min, one treatment cycle corresponding to three infusions. Patients were treated until progression. Safety follow-up was 1 month.

The starting dose of 0.48 mg kg^−1^ was determined based on nonclinical toxicology and efficacy studies (anticipated pharmacologically active dose) and subsequent conversion to human equivalent dose. This dose was anticipated to result in safety margins of tenfold compared with the predicted minimum efficacious dose.

An accelerated titration design was used. Following the first two DLs with one patient each, the 3 + 3 design was applied. The first patient at each DL was monitored for the first week of C1; if considered safe, the other patients in the cohort were treated simultaneously. The initial plan was to test five DLs and enroll six patients in DL5. Because no DLTs (excepting one grade 2 pancreatitis) were seen, it was agreed with the SMC to add a further DL—that is, DL6. However, starting at DL5 an increased number of IRRs were observed and tissue saturation was detected (PK curve), and this was also confirmed at DL6. It was therefore decided to concentrate on DL5, which was then backfilled and chosen as the RP2D.

Note that the original study design also included an amendment (no. 3) to enroll double-hit lymphoma (DHL) patients; however, this amendment was considered only at a much later time compared with the start of the trial because enrollment of DHL patients was planned for phase 2. Because the phase 2 part of the study was canceled, no DHL patients were eventually enrolled in phase 1.

Tumor biopsy (from either the primary tumor or metastases) had to be obtained from patients during both screening and treatment, following at least 2 weeks of treatment and three infusions (at around C1D15).

The study was conducted according to the principles of Good Clinical Practice and the Declaration of Helsinki, and approvals from the Country Competent Authority and Central Ethics Committee were obtained before starting any study-related procedures. The study was registered according to country-specific regulations.

Safety monitoring committee members were responsible for safeguarding the interests of patients, assessing the safety of the study drug during the study and any study-specific interventions required. The committee reviewed toxicity and other relevant data at the completion of each DL cohort and issued a recommendation pertaining to dose escalation. Intrapatient dose escalation was allowed when a higher dose was considered safe by the SMC.

### Outcomes

The primary objective of the study was to determine the safety and tolerability of single-agent OMO-103. Secondary objectives were RP2D, PK, PD and preliminary antitumor activity, including identification of potential biomarkers.

Dose-limiting toxicity, MTD and RP2D: Toxicities were graded according to the Common Terminology Criteria for Adverse Events (NCI–CTCAE) v.5.0. A DLT was defined as a grade 3 or higher adverse event according to NCI–CTCAE, considered at least possibly related to the study drug and occurring during the 21-day observation period. MTD was defined as the DL below that associated with DLTs in either two out of three or two out of six patients. MTD was not reached in this study.

RP2D was selected based on clinical safety/tolerability, antitumor activity, PK and PD data.

Clinical response was assessed using CT according to RECIST (v.11) criteria at pretreatment and every 9 weeks thereafter.

### ctDNA assay

Blood samples for exploratory biomarker ctDNA analyses were obtained and subjected to the next-generation sequencing panel Guardant360 at Guardant Health, Inc. The Guardant360 assay detects single-nucleotide variants, indels, fusions and copy number alterations in 73 genes^36^. All plasma specimens collected at (1) baseline (except for one patient that was removed from the study due to brain metastases), were genotyped (*n* = 21) when available at C2D1 (*n* = 18) and selected at C4 and/or C6 (*n* = 7) and at (2) progression (*n* = 3) (Supplementary Table 1).

### Volumetric tumor assessment

All contrast-enhanced CT scans were acquired within 28 days before the treatment starting day, at 9 weeks of treatment (that is, after three cycles) and then every 9 weeks while on treatment. We further explored responses by changes in volume in those patients achieving SD/partial response/complete response as best response according to RECIST v.1.1. All tumors (primary and metastatic disease) of at least 1 cm in diameter (that is, considered measurable according to RECIST v.1.1) per patient were segmented using the semiautomatic segmentation tool of 3DSlicer (v.4.11.0)^37^ by an experienced radiologist in oncologic imaging (R.P.-L.). Pixel size and number were recorded to calculate total tumor volume (pixel size × number) per patient and compute changes in volume from baseline.

### PK and ADAs

Serum samples for PK assessment were taken at C1, 3, 6 and 9, just before dosing, and at 5 min, 30 min, 1 h, 2 h, 6 h, 24 h, 48 h, 72 h and 96 h following the end of infusion (the time points of 48, 72 and 96 h were applied only to patients from DL3 onwards). Serum was isolated using standard procedures and cryopreserved at −80 °C until use. Concentrations of OMO-103 were determined using a validated electrochemiluminescence assay (ECLA) method. PK parameters (*t*1/2, AUClast, AUCinf, AUCinf/D, Vz and Cl) were estimated using noncompartmental analysis following single and multiple administrations.

Serum samples for ADA assessment were taken at screening, before dosing at C1, 2 and 3 and then every three cycles, and at the end of both treatment and follow-up. Serum was isolated using standard procedures and cryopreserved at −80 °C until use. The presence of anti-Omomyc ADAs was measured using a validated ECLA method performed according to the bioanalytical guidelines of the European Medicines Agency and Food and Drug Administration. The analytical method consisted of an ECL assay in a bridging format. Serum samples were incubated in the presence of biotin-Omomyc and sulfoTAG-Omomyc and the resulting immune complexes (biotin-Omomyc/anti-Omomyc antibodies/sulfoTAG-Omomyc) were captured using a streptavidin plate and measured by ECLA.

Both PK and ADA assays were conducted in compliance with the Principles of Good Laboratory Practices regulations.

### DSP GeoMx Human Whole Transcriptome Atlas

Paired formalin-fixed, paraffin-embedded (FFPE) biopsies collected before and during treatment (Extended Data Table 2) were sectioned and stained for PANCK to identify tumor cells. Samples were prepared according to tumor type following Nanostring’s procedure then loaded into the DSP instrument for selection of between one and five polygonal regions of interest (500–600 µm per sample).

Digital spatial profiling data were analyzed using the GeoMxTools R package (v.3.0.1). Quality check and preprocessing of the data were performed using the standard parameters as suggested by Nanostring (https://github.com/Nanostring-Biostats/GeomxTools). Quartile 3 normalization was used to normalize data. For differential gene expression analysis within patient samples (pre- and on-treatment biopsies) we used linear mixed-effect models with the functions provided by GeoMxTools, while comparisons between groups of patients (SD and PD) were performed using the limma R package (v.3.52.4). GSEA was conducted using the clusterProfiler R package (v.4.4.4). MYC gene sets were obtained from the chemical and genetic perturbations collection, Biological Process ontology gene sets were obtained from the Gene Ontology collection and hallmark gene sets were obtained from the Hallmark collection, all in the molecular signatures database. Data were plotted using the ggplot2 R package (v.3.3.6).

### Pharmacodynamics

Blood samples were taken at pretreatment (just before patients received the first OMO-103 infusion and before every subsequent infusion) and at 1, 2 and 6 h following infusion. Serum was then isolated using standard procedures and cryopreserved at −80 °C until use. Levels of cytokines, chemokines and other soluble factors were measured by the Luminex technique using the Inflammation 20-Plex Human ProcartaPlex kit (Invitrogen) that includes GM-CSF, IFNα, IFNγ, IL-1α, IL-1β, IL-4, IL-6, IL-8, IL-10, IL-12p70, IL-13, IL-17A, TNFα, IP-10, MCP-1, MIP-1α, MIP-1β, ICAM-1, CD62E (E-selectin) and CD62P (P-selectin). Plates were read using the MAGPIX detection system. Soluble factor levels were extrapolated from standard curves using ProcartaPlex Analysis Software v.2.2.0. For analysis of the predictive and pharmacodynamic signature, only patients evaluable for response were considered.

### MYC immunohistochemical analysis

For evaluation of MYC expression, pretreatment (*n* = 18) and on-treatment (*n* = 13) biopsies from 19 patients were analyzed using 100 μl of a ready-to-use solution of anti-c-MYC (Y69) rabbit monoclonal primary antibody (Roche) per sample, following the protocol described in ref. ^38^. Briefly, staining was performed using a Benchmark ULTRA autostainer (Ventana Medical Systems) and detection was carried out with the UltraView Universal DAB Detection kit (no. 760–500, Ventana Medical Systems). For assessment of MYC a semiquantitative approach was used in which *H*-scores were generated by multiplying staining intensity (0, no staining; 1, weak; 2, moderate; 3, strong) by the percentage (0–100) of positive cells.

### OMO-103 quantification by LC–MS parallel reaction monitoring and ultradeep data-independent acquisition profiling in patient biopsies

Samples were prepared according to the Biognosys Suite of Proteomics for FFPE samples, including sonication, deparaffinization and homogenization, using the Covaris LE220Rsc sonication device. Samples were then processed individually on a KingFisher Flex overnight at 37 °C according to the Biognosys Suite of Proteomics, which includes reduction, alkylation and digestion of peptides using trypsin (Promega, ratio of 1:50 protease to total protein) and Lys-C (Fujifilm Wako Chemicals, ratio of 1:200 protease to total protein).

Clean-up for MS was carried out using an Oasis HLB μElution plate (30 μm, Waters) according to the manufacturer’s instructions. Peptides were dried to complete dryness using a SpeedVac system and dissolved in LC solvent A (1% acetonitrile in water and 0.1% formic acid (FA)) containing Biognosys indexed retention time peptide mix for retention time calibration. Peptide concentrations in MS-ready samples were measured using the mBCA assay (Thermo Scientific Pierce).

For OMO-103 quantification, five stable isotope-labeled reference peptides were spiked into the final peptide samples at known concentrations (Vivitide; the quality grade of the reference peptides was ±10% quantification precision and >95% purity).

For LC–MS parallel reaction monitoring (LC–PRM) measurement, 1 μg of peptides per sample was injected into an in-house-packed C18 column (PicoFrit emitter with 75 μm inner diameter, 60 cm length and 10 μm tip from New Objective, packed with 1.7 μm of Charged Surface Hybrid C18 particles from Waters) on a Thermo Scientific Easy nLC 1200 nanoLC system connected to a Thermo Scientific Q Exactive mass spectrometer equipped with a standard nanoelectrospray source. LC solvents were: A, 1% acetonitrile in water with 0.1% FA and B, 20% water in acetonitrile with 0.1% FA. The LC gradient was 0–59% solvent B over 54 min followed by 59–90% B for 12 s and 90% B for 8 min (total gradient length 67 min). A scheduled run in PRM mode was performed before data acquisition for retention time calibration using the Biognosys indexed retention time concept^39^. The data acquisition window per peptide was 7 min. Signal processing and data analysis were carried out using SpectroDive 11.0—Biognosys’ software for multiplexed PRM data analysis based on mProphet^39,40^. A *Q*-value filter of 1% was applied.

For ultradeep data-independent acquisition (DIA) LC–tandem MS measurements, 2 µg of peptides or all peptides (Biognosys ID 3, 4 and 7) was loaded on an in-house-packed reversed-phase column of a Thermo Scientific NeoVanquish UHPLC nanoLC system connected to a Thermo Scientific Orbitrap Exploris 480 mass spectrometer equipped with a Nanospray Flex ion source and a FAIMS Pro ion mobility device (Thermo Scientific). LC solvents were: A, water with 0.1% FA and B, 80% acetonitrile and 0.1% FA in water. The nonlinear LC gradient was 1–50% solvent B for 172 min followed by a column-washing step in 90% B for 5 min, and a final equilibration step of 1% B for one column volume with flow rate set to a ramp of 500–250 nl min^−1^ (min 0, 500 nl min^−1^; min 172, 250 nl min^−1^, washing at 500 nl min^−1^). The FAIMS DIA method consisted of an applied compensation voltage of one full-range MS1 scan and 34 DIA segments, as previously adopted^41,42^.

The DIA MS data were analyzed using directDIA+ in Spectronaut software (Biognosys, v.18.4) with the false discovery rate for peptide and protein levels set to 1%. A human UniProt.fasta database (*Homo sapiens*, 2023-07-01) was used for the search engine, allowing for two missed cleavages and variable modifications (N-term acetylation, methionine oxidation and methylation) and cysteine carbamidomethylation as fixed modification. The assay library (protein inventory) generated in this project was used for analysis. The hyper reaction monitoring measurements analyzed with Spectronaut were normalized using local regression normalization^43^.

### Statistical methods and analysis

Sample size was determined based on clinical rather than statistical considerations. Twenty-two patients were recruited, this being consistent with phase 1 dose-finding studies.

Statistical analyses were performed by the Clinical Research Organization Simbec-Orion using SAS v.9.4 or later (SAS Institute).

Safety and tolerability were analyzed through the incidence of AEs, serious AEs and specific laboratory abnormalities (worst grade) in each DL. Toxicities were tabulated by type and grade for all doses and presented using frequencies based on CTCAE v.5.0. All subjects who received at least one dose of the study drug were evaluable for safety (safety analysis set).

Antitumor activity was assessed using RECIST v.1.1 and included ORR, progression-free survival and disease control rate. All efficacy endpoints were summarized and analyzed descriptively using the full analysis set (all patients in the SAF who completed at least one follow-up assessment, defined as any primary assessment variable recording using RECIST v.1.1).

PK analyses were performed from experimental data and using actual sampling times and dosing levels of each subject via the PKSolver add-in program for Microsoft Excel, by means of a noncompartmental approach.

### Predictive signatures of soluble factors

To determine whether the levels of the examined soluble factors were different between patients with PD and SD at pretreatment, a univariate analysis was performed. A two-sided Mann–Whitney U-test with no adjustment for multiple comparisons was used to determine whether the levels of soluble factors between the two groups were significantly different. To calculate OR the levels of soluble factors were transformed using the log_2_ scale. Individual (univariate) binary logistic regression models, in which the exponential of the coefficients can be directly interpreted as OR, were applied using the statistical software package SPSS statistics (SPSS). Logistic regression was used to measure the strength of the association between levels of soluble factors at baseline and response to OMO-103 treatment. The diagnostic accuracy of the individual soluble factors in identifying patients with SD was estimated using ROC–AUC, which was also used as a global measure to compare the predictive power of each individual soluble factor. A cutoff of AUC = 0.8 was used for selection of soluble factors with good predictive power. The analysis was performed by Abzu fitting of a logistic regression model to each soluble factor individually, with RECIST v.1.1 evaluation at C3 as the binary output variable. The Python package Scikit-learn was used to fit the models^44^.

Combinations of soluble factors that can correctly stratify patients between PD and SD were found using QLattice modeling technology (Abzu)^45^. QLattice was run within a leave-one-out cross-validation loop, excluding a single patient at each iteration. ROC–AUC was also calculated for the combination models to determine its prediction accuracy and power. Confidence interval bands of the models were estimated using the parameter values of all models found.

### Pharmacodynamic/response signatures of soluble factors

To determine whether the increase in the serum levels of various soluble factors was different between SD and PD patients at the onset of C3, their levels relative to pretreatment of this particular cycle were calculated. A total of ten patients were considered in this analysis (six SD and four PD); one patient showing the signature at C6 rather than at C3 was excluded from the analysis. *P* value was used to reject the null hypothesis. A two-sided Welch’s *t*-test was used to determine whether the levels of cytokines, chemokines and other soluble factors between the two groups were significantly different. Bonferroni correction was used to adjust for multiple comparisons. Again, QLattice modeling technology (Abzu) was used to determine single models that could estimate the soluble factor levels that distinguish between patients with SD and PD. To generate the models, for each patient the levels of only one time point (the maximum level of each soluble factor) were considered. QLattice was run within a leave-one-out cross-validation loop, excluding a single patient at each iteration. ROC–AUC was also calculated for the single models to determine their prediction accuracy and power (as described above). Confidence interval bands of the models were estimated using the parameter values of all models found.

The datasets generated during and/or analyzed during the current study, as well as the custom codes used for its analyses, are available from the corresponding author on reasonable request.

### Reporting summary

Further information on research design is available in the Nature Portfolio Reporting Summary linked to this article.

## Online content

Any methods, additional references, Nature Portfolio reporting summaries, source data, extended data, supplementary information, acknowledgements, peer review information; details of author contributions and competing interests; and statements of data and code availability are available at 10.1038/s41591-024-02805-1.

### Supplementary information

Reporting Summary

Supplementary Tables 1 and 2.

### Source data

Source Data Fig. 1 Stastistical source data.

Source Data Fig. 2 Stastistical source data.

Source Data Fig. 5 Stastistical source data.

Source Data Fig. 6 Stastistical source data.

Source Data Extended Data Table 1 Stastistical source data.

Source Data Extended Data Table 2 Stastistical source data.

## Figures and Tables

**Extended Data Table 1:**
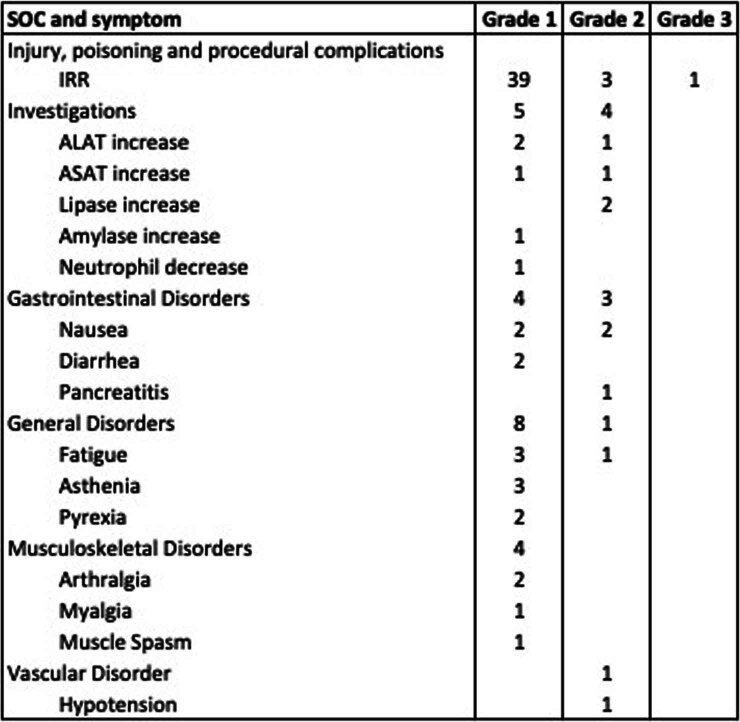
Treatment-related adverse events (TRAEs) with severity grade according to the Common Terminology Criteria for Adverse Events version 5.0

Reported System Organ Class (SOC) and symptoms are shown with the numbers of events. IRR: Infusion Related Reaction; ALAT: Alanine-Aminotransferase; ASAT: Aspartate-Aminotransferase.

Source data

**Extended Data Table 2:**
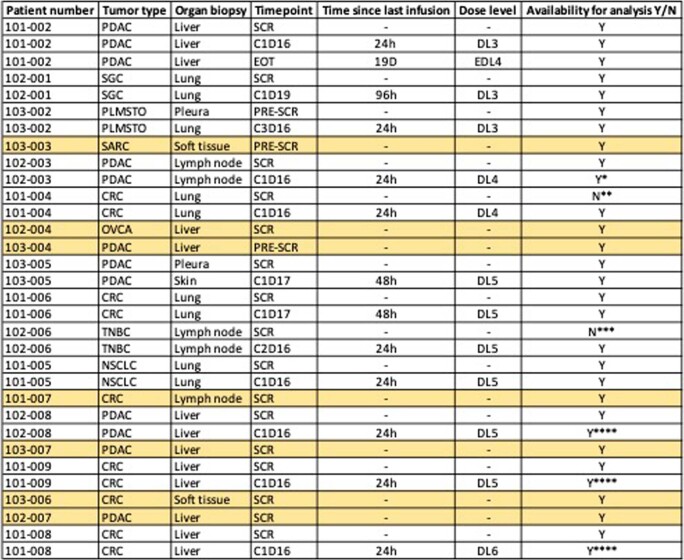
Timepoints for biopsy collection and availability

SCR: screening; PRE-SCR: pre-screening (up to 6 months before entering the study); CXDX: cycle x day x; DL: dose level. Colored in yellow are the patients from whom we only received the pre-treatment biopsy. Y/N: yes or no. * biopsy was lost during DSP processing. ** biopsy did not have tumor tissue. *** biopsy material used up with IHC assessment. **** not evaluated by DSP.

Source data

**Extended Data Table 3:**
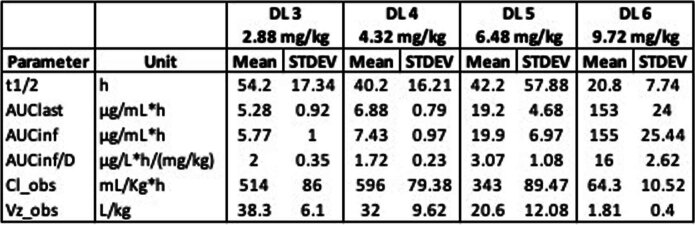
Summarized descriptive statistics of the pharmacokinetic parameters of OMO-103 after multiple intravenous infusions of OMO-103 with different dose levels

PK parameter statistics were not calculated for DL 1 and 2, where only one patient was treated. The mean and standard deviation (STDEV) values are shown. t1/2: Terminal half-life; AUClast: Area Under the serum concentration-time Curve within a dosage interval (0 to last measurable concentration) after the first dose; AUCinf: AUC from time of dosing extrapolated to infinity, based on the last predicted concentration; AUCinf/D: AUC from time of dosing extrapolated to infinity, relative to the dose; Cl: Total body clearance; Vz: Volume of distribution based on the terminal phase.

**Extended Data Table 4:**
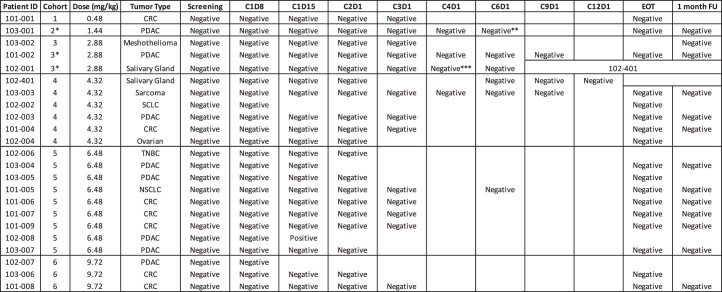
Detection of Anti-Drug Antibodies (ADAs) in serum over time

The presence or absence of ADAs, quantified as positive or negative, at the indicated time points during the OMO-103 Phase I clinical trial is shown. Patient 102-001 ID changed to 102-401 when it was escalated to the next dose level. CRC: colorectal cancer; PDAC: pancreatic ductal adenocarcinoma; SCLC: Small Cell Lung Cancer; TNBC: Triple-Negative Breast Cancer; NSCLC: non-small-cell lung cancer. *Escalated to next dose level; **C6D8; ***C4D15.

**Extended Data Table 5:**
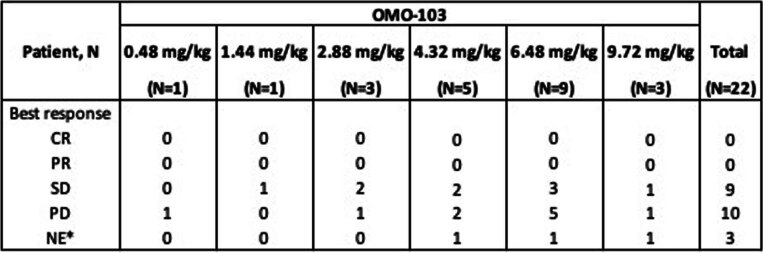
Summary table for all patients and their efficacy results according to RECIST

CR complete response; PR partial response; SD stable disease; PD progressive disease; NE not evaluable; *No second assessment. Responses according to RECIST 1.1 by CT scan.

## Data Availability

The raw data for DSP analysis are available at 10.5281/zenodo.10420686. On reasonable request, and subject to review, Peptomyc will provide the multiplex data that support the findings of this study. Subject to certain criteria, conditions and exceptions, Peptomyc may also provide access to the related individual deidentified participant data. The protocol and statistical analysis plan for MYCure have been uploaded to ClinicalTrials.gov. Source data are provided with this paper.
