# Supplementary material for: MYC targeting by OMO-103 in solid tumors: a phase 1 trial
Source: Nat Med. 2024 Feb 6;30(3):762–71. doi: 10.1038/s41591-024-02805-1 (PMC10957469; doi:10.1038/s41591-024-02805-1)
Supplement: Supplementary file 1 — Reporting Summary [file 41591_2024_2805_MOESM1_ESM.pdf]

nature portfolio

Corresponding author(s): Laura Soucek

Last updated by author(s): Dec 7, 2023

Reporting Summary

Nature Portfolio wishes to improve the reproducibility of the work that we publish. This form provides structure for consistency and transparency in reporting. For further information on Nature Portfolio policies, see our Editorial Policies and the Editorial Policy Checklist.

Statistics

For all statistical analyses, confirm that the following items are present in the figure legend, table legend, main text, or Methods section.

n/a	Confirmed	
☐	☒	The exact sample size (*n*) for each experimental group/condition, given as a discrete number and unit of measurement
☐	☒	A statement on whether measurements were taken from distinct samples or whether the same sample was measured repeatedly
☐	☒	The statistical test(s) used AND whether they are one- or two-sided<br>*Only common tests should be described solely by name; describe more complex techniques in the Methods section.*
☒	☐	A description of all covariates tested
☐	☒	A description of any assumptions or corrections, such as tests of normality and adjustment for multiple comparisons
☐	☒	A full description of the statistical parameters including central tendency (e.g. means) or other basic estimates (e.g. regression coefficient) AND variation (e.g. standard deviation) or associated estimates of uncertainty (e.g. confidence intervals)
☐	☒	For null hypothesis testing, the test statistic (e.g. *F*, *t*, *r*) with confidence intervals, effect sizes, degrees of freedom and *P* value noted<br>*Give P values as exact values whenever suitable.*
☒	☐	For Bayesian analysis, information on the choice of priors and Markov chain Monte Carlo settings
☒	☐	For hierarchical and complex designs, identification of the appropriate level for tests and full reporting of outcomes
☒	☐	Estimates of effect sizes (e.g. Cohen's *d*, Pearson's *r*), indicating how they were calculated

Our web collection on statistics for biologists contains articles on many of the points above.

Software and code

Policy information about availability of computer code

Data collection	Soluble factors levels were extrapolated from the standard curves using the ProcartaPlex Analyst Software.
Data analysis	DSP data was analyzed using the GeoMxTools R-package (version 3.0.1). Comparisons between groups of patients (stable and progressive disease) were done using the limma R-package (version 3.52.4). Gene Set Enrichment Analysis (GSEA) was conducted using the clusterProfiler R-package (version 4.4.4). Data was plotted using the ggplot2 R-package (version 3.3.6). Soluble factors levels were extrapolated from the standard curves using the ProcartaPlex Analyst Software (version 2.2.0) and plotted using the Graphpad Prism software (version 8). Clinical Statistical analyses were performed by the Clinical Research Organization Simbec-Orion using SAS®, Version 9.4 or later, SAS Institute, Cary, Northern Carolina, USA. The PK analyses were performed from experimental data and using actual sampling times and actual dosing levels of each subject using the PKSolver add-in program for Microsoft Excel by means of a non-compartmental approach. For soluble markers analysis, the Python package Scikit-learn was used to fit the models. Combinations of soluble factors that can correctly stratify patients between PD and SD were found using the QLattice modelling technology (Abzu). Data analysis of OMO-103 quantification from patient biopsies were carried out using SpectroDive™ 11.0 – Biognosys' software for multiplexed PRM data analysis.The DIA mass spectrometric data were analyzed using directDIA+ in Spectronaut software (Biognosys, version 18.4).A human UniProt .fasta database (Homo sapiens, 2023-07-01) was used for the search engine. For volumetric tumor assessment, all those considered measurable by RECIST v1.1 were segmented using the semi-automatic segmentation tool of 3DSlicer (version 4.11.0).

For manuscripts utilizing custom algorithms or software that are central to the research but not yet described in published literature, software must be made available to editors and reviewers. We strongly encourage code deposition in a community repository (e.g. GitHub). See the Nature Portfolio guidelines for submitting code & software for further information.

Data

Policy information about availability of data

All manuscripts must include a data availability statement. This statement should provide the following information, where applicable:

- Accession codes, unique identifiers, or web links for publicly available datasets
- A description of any restrictions on data availability
- For clinical datasets or third party data, please ensure that the statement adheres to our policy

The datasets generated during and/or analysed during the current study used for its analyses are available from the corresponding author on reasonable request. This has been stated in the text.

Human research participants

Policy information about studies involving human research participants and Sex and Gender in Research.

Reporting on sex and gender	This Phase 1 study was open to all patients, regardless of sex, who fulfilled the inclusion criteria. There was also no age limit above 18 years old.
Population characteristics	The study included histologically or cytologically proven advanced solid tumours, for which there was no curative therapy, that had progressed on standard of care (SOC) treatment, were intolerant to it or had no available SOC or for which SOC was unacceptable. Patients had to display measurable disease as per RECIST v1.1 criteria 35 proven by CT/MRI and documented progression on or following the last line of therapy. They also had to present an Eastern Cooperative Oncology Group (ECOG) performance status of up to 1, life expectancy of ≥12 weeks and adequate organ function. Sex was equally distributed between patients; median age was 60.5 years . They were all caucasian and had received between 2 and 12 previous treatments (median 4 treatments).
Recruitment	Patients were selected by very experienced Phase 1 Investigators at 3 sites in Spain; patients had to fulfill all required criteria; ECOG had to be either 0 or 1 and other strict criteria had to be fulfilled (see paragraph above fo details).
Ethics oversight	The study was approved by the ethics committee of the Vall d'Hebron Hospital, Barcelona. However, the final approval for the whole study came from the Spanish "AEMPS". The study was conducted according to the principles of Good Clinical Practice and the Declaration of Helsinki The EC also approved the informed consent form which was used in the clinical trial.

Note that full information on the approval of the study protocol must also be provided in the manuscript.

Field-specific reporting

Please select the one below that is the best fit for your research. If you are not sure, read the appropriate sections before making your selection.

☒ Life sciences ☐ Behavioural & social sciences ☐ Ecological, evolutionary & environmental sciences

For a reference copy of the document with all sections, see nature.com/documents/nr-reporting-summary-flat.pdf

Life sciences study design

All studies must disclose on these points even when the disclosure is negative.

Sample size	As this is a typical Phase 1 study, there is no sample size calculation. An accelerated titration design was used. After the first 2 dose levels with 1 patient each, the 3+3 design was applied, and dose escalation was done until dose level 6.
Data exclusions	As per the safety study, all patients who received the study drug at least once are included into the safety analysis as described in the SAP.
Replication	Not applicable for this Phase 1 study
Randomization	This was a Phase 1 dose escalation study with single agent OMO-103, thus no randomisation was necessary nor applicable.
Blinding	This was a Phase 1 dose escalation study with single agent OMO-103, thus no blinding was necessary nor applicable.

Reporting for specific materials, systems and methods

We require information from authors about some types of materials, experimental systems and methods used in many studies. Here, indicate whether each material, system or method listed is relevant to your study. If you are not sure if a list item applies to your research, read the appropriate section before selecting a response.

Materials & experimental systems

n/a	Involved in the study
☐	☒ Antibodies
☒	☐ Eukaryotic cell lines
☒	☐ Palaeontology and archaeology
☒	☐ Animals and other organisms
☐	☒ Clinical data
☒	☐ Dual use research of concern

Methods

n/a	Involved in the study
☒	☐ ChIP-seq
☒	☐ Flow cytometry
☒	☐ MRI-based neuroimaging

Antibodies

Antibodies used

anti-c-MYC (Y69) Rabbit Monoclonal Primary Antibody (Cat#790-4628) Roche

Validation

INTENDED USE
Anti-c-MYC (Y69) Rabbit Monoclonal Primary Antibody is intended for laboratory use in the qualitative immunohistochemical detection of c- MYC protein by light microscopy in sections of formalin-fixed, paraffin- embedded tissue stained on a BenchMark IHC/ ISH instrument. This product should be interpreted by a qualified pathologist in conjunction with histological examination, relevant clinical information, and proper controls. This antibody is intended for in vitro diagnostic (IVD) use.

Clinical data

Policy information about clinical studies
All manuscripts should comply with the ICMJE guidelines for publication of clinical research and a completed CONSORT checklist must be included with all submissions.

Clinical trial registration

NCT04808362; EudraCT No 2020-003802-30

Study protocol

attached as appendix

Data collection

A commercially available eCRF system was used for data collection: Oracle Inform; the access to the system was limited to authorised personnel only. The first patient was enrolled on the May 4th 2021 and the last patient on April 4th 2022;. The recruitment lasted11 months and the last patient came off study January 11th 2023. Site staff entered the data continuously into the system; throughout the study. These entries were controlled by programmed checks of the system but also by the CRAs of the CRO.

Outcomes

Primary endpoint: safety via:
• Number of patients with a DLT;
• Number of patients with ≥1 related AE;
• Number of patients discontinuing study treatment due to related AEs
Secondary endpoint: efficacy via CT-imaging according to RECIST eg ORR, PFS, DCR, DOR
all AEs and SAEs were collected in the eCRF and later on extracted and evaluated by the statistical team, and methods applied by number, grade and relationship to study drug.
Secondary endpoint: efficacy is assessed via pre-planned CT scans, which were evaluated by experienced radiologists according to the well known and validated RECIST criteria. From the responses, several other parameters were derived according to the statistical analysis plan (eg PFS, DCR, DOR). PK was assessed by a specialised laboratory according to the validated method. A specific statistical program was used to analyse the PK data.
