# Supplementary material for: MYC targeting by OMO-103 in solid tumors: a phase 1 trial
Source: Nat Med. 2024 Feb 6;30(3):762–71. doi: 10.1038/s41591-024-02805-1 (PMC10957469; doi:10.1038/s41591-024-02805-1)
Supplement: Supplementary file 2 — Supplementary Tables 1 and 2. [file 41591_2024_2805_MOESM2_ESM.pdf]

Supplementary Table 1: Genomic landscape of patient ctDNA. Individual patient results are shown in rows, and cfDNA identified alterations are shown in each column. All patients had multiple samples analyzed, referred to as the timepoint in which they were collected. The variant allele frequency (VAF) is shown in each cell only when their percentage is ≥ 0.3% for, at least, one of the timepoints. Patients that presented more than one variant per gene have their percentages listed in the same order for all timepoints. When missing in one or another timepoint a blank is left. Color legend: blue shade, MYC variants; blue, single nucleotide variants (SNV); green, indels (insertions or deletions); red, copy number variants (CNV) and amplifications (AMP). NA: not applicable; SCR: screening; CXDX: cycle x day X; EOT: end of treatment.

Alteration type	Timepoint	MYC E425T	MYC I143I	MYC CNV	CDK12 SNV	TP53 SNV	APC SNV	SMAD4 SNV	ATM SNV	KRAS SNV	PIK3CA SNV	CDK4 SNV	EGFR SNV	AR SNV	STK11 SNV	PDGFRA SNV	NOTCH1 SNV	FBXW7 SNV	BRCA2 SNV	ERBB2 SNV	CDK6 SNV	NTRK1 SNV	FGFR2 SNV	MTOR SNV	FGFR3 SNV	TP53 Indel	APC Indel	ATM Indel	ARID1A Indel	MAPK3 Indel	CDKN2A Indel	MAP2K1 Indel	SMAD4 Indel	BRCA2 Indel	FGFR1 AMP	CCND2 CNV	PIK3CA CNV	BRAF CNV	RAF1 CNV	EGFR CNV	KRAS CNV	CCNE1 AMP	MET CNV	CCND1 CNV	ERBB2 CNV
# of pts	1	0	1	0	2	6	5	3	1	11	4	1	2	2	2	1	1	1	2	1	1	1	1	1	0	2	5	1	1	1	1	0	0	0	2	1	1	1	1	0	0	0	0	0	0
	2	1	1	1	2	6	6	3	1	11	4	1	2	3	2	1	0	1	3	0	1	1	0	0	1	2	5	1	1	1	1	1	1	1	2	1	1	2	0	1	1	2	1	1	1
101-001	SCR				0,53																					60,97	12,31								2,92										
	EOT				0,65									0,75					0,84						0,31	82,49	24,61					1,33			3,6			2,51		2,49					
101-002	SCR							0,33																			0,9																		
	C6D1							1,85																			5,56																		
102-001	SCR																											0,31																	
	C4D15																											0,23																	
101-004	SCR						2,78			2,62	1,31	0,42	0,28													2,74																			
	C2D1						17,62			13,94	7,07	0,41	1,19													16,81																			
102-003	SCR					3,06				1,42																																			
	C2D1	0,36				4,9				1,33																																			
102-004	SCR		0,82						2,01<br>0,31				4,69<br>3,2	1,14<br>0,37																						2,69	2,63	2,55	2,33						
	C2D1		0,57						0,82<br>0,33				2,74<br>3,65	1,3																							2,4								
	EOT		1,72				1,16		0,67				7,15<br>2,08	0,82																						2,67	3,18	2,6			2,62	2,43	2,28		
101-005	SCR					6,83				4,38					6,78<br>6,57																														
	C6D1					13,38				9,19					10,24<br>9,85																														
101-006	SCR						3,97			7,13				14,34		0,4											5,66		5,22	1,11															
	C2D1						3,85			6,07				13,82		0,39											4,64		4,8	1,09															
101-007	C1D1						2,08			2,36	2,02						0,33	0,71									1,26																		
	C4D1						1,27			2,71	1,47							0,55									1																		
102-006	SCR							1,01<br>0,54<br>0,49<br>0,38<br>0,54											2,38	1,04	0,45														3,1										
	C2D1							0,92<br><br>0,51<br>0,45<br><br>0,68											3,02<br>0,42		0,19												0,36		3,11									3,51	
103-004	SCR					3,42<br>0,41				3,21												0,92																							
	EOT			3,01		11,31<br>0,37				9,39												0,83																							2,33
103-005	SCR					4,36		1,93		6,02																					3,17														
	C2D1					4,25		1,01		5,81																					3,24														
103-007	SCR				0,67					0,61					0,94				1,1																										
	C2D1				2,59					2,82					3,48				4,71															0,93											
101-009	SCR						4,8			2,98	6,35																																		
	C2D1						6,77			4,69	7,3																																		
103-006	SCR					17,81<br>15,72	15,74<br>8,68<br>0,58			12,88	14,77												0,66	0,63																					
	C2D1					8,81<br>8,34	9,41<br>5,71			8,04	8,71																															2,29			
101-008	C1D1					0,55				1,27																	0,47																		
	C4D1					2,15				3,14																	0,94																		

Supplementary Table 2: SD patients display a specific pharmacodynamic signature. Characteristics of the patients used for the pharmacodynamic signature are shown. 102-001 displayed the pharmacodynamic signature at C6 instead of C3. PDAC: pancreatic ductal adenocarcinoma; NSCLC: non-small cell lung cancer; CRC: colorectal cancer.

Patient ID	Cohort	Dose (mg/kg)	Tumor type	Timepoint	Response RECIST 1.1	Pharmacodynamic signature
102-001	3	2,88	Salivary Gland	C6*	SD	YES
101-002	3	2,88	PDAC	C3	SD	YES
103-003	4	4,32	Sarcoma	C3	SD	YES
101-005	5	6,48	NSCLC	C3	SD	YES
101-007	5	6,48	CRC	C3	SD	YES
101-008	6	6,48	CRC	C3	SD	YES
101-009	5	9,72	CRC	C3	SD	YES
103-002	3	2,88	Mesothelioma	C3	PD	NO
101-004	4	4,32	CRC	C3	PD	NO
102-003	4	4,32	PDAC	C3	PD	NO
101-006	5	6,48	CRC	C3	PD	NO
